# Protein intake *during* training sessions has no effect on performance and recovery during a strenuous training camp for elite cyclists

**DOI:** 10.1186/s12970-016-0120-4

**Published:** 2016-03-05

**Authors:** Mette Hansen, Jens Bangsbo, Jørgen Jensen, Matilde Krause-Jensen, Bo Martin Bibby, Ove Sollie, Ulrika Andersson Hall, Klavs Madsen

**Affiliations:** Section of Sport Science, Department of Public Health, Aarhus University, Dalgas Avenue 4, 8000 Aarhus C, Denmark; Department of Nutrition, Exercise and Sports, University of Copenhagen, Copenhagen, Denmark; Department of Physical Performance, Norwegian School of Sport Science, Oslo, Norway; Section for Biostatistics, Department of Public Health, Aarhus University, Aarhus, Denmark; Department of Food and Nutrition, and Sport Science, University of Gothenburg, Gothenburg, Sweden

**Keywords:** Athletes, Muscle damage, Creatine kinase, Power, Endurance performance

## Abstract

**Background:**

Training camps for top-class endurance athletes place high physiological demands on the body. Focus on optimizing recovery between training sessions is necessary to minimize the risk of injuries and improve adaptations to the training stimuli. Carbohydrate supplementation during sessions is generally accepted as being beneficial to aid performance and recovery, whereas the effect of protein supplementation and timing is less well understood. We studied the effects of protein ingestion during training sessions on performance and recovery of elite cyclists during a strenuous training camp.

**Methods:**

In a randomized, double-blinded study, 18 elite cyclists consumed either a whey protein hydrolysate-carbohydrate beverage (PRO-CHO, 14 g protein/h and 69 g CHO/h) or an isocaloric carbohydrate beverage (CHO, 84 g/h) during each training session for six days (25–29 h cycling in total). Diet and training were standardized and supervised. The diet was energy balanced and contained 1.7 g protein/kg/day. A 10-s peak power test and a 5-min all-out performance test were conducted before and after the first training session and repeated at day 6 of the camp. Blood and saliva samples were collected in the morning after overnight fasting during the week and analyzed for biochemical markers of muscle damage, stress, and immune function.

**Results:**

In both groups, 5-min all-out performance was reduced after the first training session and at day 6 compared to before the first training session, with no difference between groups. Peak power in the sprint test did not change significantly between tests or between groups. In addition, changes in markers for muscle damage, stress, and immune function were not significantly influenced by treatment.

**Conclusions:**

Intake of protein combined with carbohydrate during cycling at a training camp for top cyclists did not result in marked performance benefits compared to intake of carbohydrates when a recovery drink containing adequate protein and carbohydrate was ingested immediately after each training session in both groups. These findings suggest that the addition of protein to a carbohydrate supplement consumed during exercise does not improve recovery or performance in elite cyclists despite high demands of daily exhaustive sessions during a one-week training camp.

## Background

During periods of high-intensity training, nutrition is of outmost importance for top athletes in order to optimize training quality, muscular adaptations, and recovery between sessions. Similarly, endurance races over several days necessitate fast recovery. It is generally accepted that ingestion of carbohydrate is beneficial for performance and recovery during high-intensity endurance sports [1, 2]. The effect of protein on endurance performance and recovery is less well understood. The majority of studies on protein supplementation during exercise show no acute performance effect [3–6], but a few studies have shown improved performance [7–10]. In most of these studies beverages were matched for carbohydrate content rather than energy [7, 9, 10] and/or a suboptimal carbohydrate beverage was used as a control [7–10], making it difficult to differentiate between the effects of elevated intake of protein or calories.

The influence of protein supplementation during [11] or post-exercise [12–14] on recovery and subsequent performance or endurance capacity has been investigated in several short-term (<24 h) studies, of which some report advantages [11, 12], whereas others report no effect [13–15] compared to intake of carbohydrate. Long-term effects (>24 h post-exercise) are less examined and there is a lack of studies on elite athletes. Current results on well-trained athletes (max. oxygen uptake >60 ml O_2_/kg/min) are mixed. Some studies show no effect of ingesting protein-carbohydrate beverages compared to control (carbohydrate) during and/or after exercise sessions on performance measured as time to exhaustion (TTE) after two weeks intervention [16], repeat-sprint performance during six days intervention [17], or running performance during six days intervention [18]. Others show performance advantages of partial substitution of carbohydrate with protein in TTE during two weeks intervention [19], time trials (TT) after one week intervention [20, 21], or repeat-sprint performance tests after 3–4 days intervention [22, 23]. Similarly, results from intervention trials are mixed in regard to effect of protein ingestion on attenuation of muscle damage. A combined intake of protein and carbohydrate has, in some cases, shown attenuation of markers for muscle damage in well-trained athletes [17–19, 21, 23, 24], whereas others report no difference [3, 20, 25]. The conditions vary considerably between studies and there is a great need for well-controlled studies focusing specifically on the effect of protein on performance and recovery over a period including daily strenuous exercise sessions.

Optimization of performance and recovery is essential at the top level where athletes often compete several days in succession. Elite athletes also have periods, typically team training camps, where they add a substantial amount of extra training. Excessive training loads in combination with inadequate recovery can further lead to a state of overreaching or overtraining, characterized by a long-term decrement in performance capacity as well as a decline in immune function [26]. It is therefore of specific interest to study nutritional strategies in order to optimize recovery during training camps with excessive training loads. The present study was designed to re-examine the nutritional practice of the Danish national team in race cycling (U23), which currently entails consumption of carbohydrate beverages during exercise and recovery beverages containing both carbohydrate and protein post-exercise. The present study compared the effect of consuming a protein-supplemented carbohydrate beverage to an isocaloric carbohydrate beverage during each session of an intense six-day training camp for Danish elite racing cyclists at Lanzarote. Under controlled conditions with cyclists, coaches, and scientists all residing at the training campus for a full week, the aim was to determine whether ingestion of a beverage containing partial substitution of carbohydrates with protein *during* exercise would have additional performance and recovery effects beyond that of the protein already present in the recovery beverage. Both acute effects of protein supplementation during the first session as well as accumulated effects over the six-day training camp were examined. It was hypothesized that there could be advantages of ingesting protein during exercise over a training period since the training sessions were extensive and lasted up to 6 h.

## Methods

### Design

**Fig. 1:**
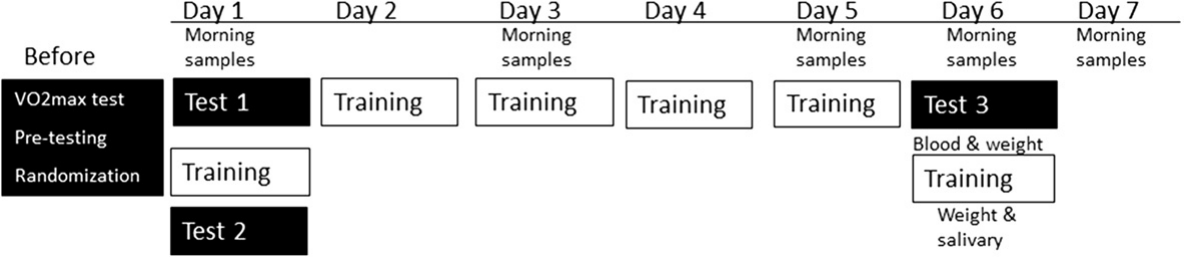
Overall design of the test protocol. Before the training camp, the cyclists performed a VO_2max_ test and were familiarized with the test protocol before the block randomization procedure. During the intervention period at the training camp (Day 1 to Day 7), the test protocol was repeated at Day 1 after breakfast, Day 1 after the training session, and Day 6 after breakfast. In the morning at Day 1, 3, 5, 6, and 7, body weight was measured, and blood samples were collected. Salivary samples were collected in the morning at Day 1, 5, 6, and 7, and 0 and 1 h after exercise at Day 6

The study was designed as a double-blinded, randomized, controlled intervention trial during a one-week training camp for elite racing cyclists (Fig. 1). The subjects were pre-tested before the camp and matched in pairs based on weight, maximal oxygen consumption (VO_2max_), a 5-min all-out performance, and training history. Afterwards they were randomized to consume either a 1) carbohydrate beverage (CHO) or a 2) protein-carbohydrate beverage (PRO-CHO) during each training session at the camp. Both groups consumed the same recovery beverage after exercise, containing 18 g protein (~0.25 g/kg) and 69 g carbohydrate (~1 g/kg). The athletes were further divided into a short distance group (~25 h/6 days) or a long distance group (29 h/6 days). The cyclists in CHO and PRO-CHO pairs were cycling the same distance.

To evaluate the acute effect of the intervention, a test protocol was performed in the morning at Day one (D1pre) and repeated after four (short distance group) or five (long distance group) hours of cycling (D1post). The test protocol was repeated at day six in the morning (D6pre). Breakfast and time points for testing at Day six were the same as at Day one of the intervention. The protocol consisted of a standardized warm-up prior to a 10-s peak power test followed by a 3-min recovery period and a 5-min all-out performance test.

Blood samples were collected before (morning sample) and immediately after the performance tests at Day 1 and Day 6 as well as in the morning of day 3, 5, and 7, and analyzed for markers of muscle damage (creatine kinase (CK), lactate dehydrogenase (LDH), and myoglobin) and cortisol. Saliva samples were collected in the morning after overnight fasting at Day 1, 5, 6, 7, immediately after exercise, and 1 h after the training session on Day 6 to be analyzed for immunoglobulin A (IgA).

### Subjects

Eighteen young, male racing cyclists (age 20 ± 2 years) were recruited for the project. All subjects were part of, or candidates to, the Danish National Team in race cycling (U23). The cyclists were block randomized to two groups, which were similar with regards to age, weight, height, fat percentage measured by the sum of four skinfold measurements [27], VO_2max_, and performance in a 5-min all-out bike test completed before the intervention period (Table 1).

**Table 1:** Subject characteristics

	CHO	PRO-CHO
Age (years)	19 ± 2	20 ± 2
Weight (kg)	71.8 ± 7.5	71.9 ± 4.2
Height (m)	1.84 ± 0.06	1.82 ± 0.04
Fat%	8.4 ± 2.3	10.0 ± 2.2
VO2_max_ (L O_2_/min)	5.2 ± 0.4	5.1 ± 0.3
Fitness level (ml O_2_/min/kg)	72.5 ± 5.2	70.9 ± 4.1
Watt_max_ (5-min Performance Test)	428 ± 32	426 ± 33

Values are mean ± SD. Fat% measured by skinfold. VO_2max_: maximal oxygen uptake. Watt_max_: Average Watt achieved in a 5-min Performance Test. No significant differences were observed between groups (*p* > 0.05) in the shown parameters

### Ethics, consent, and permissions

The study complied with the Declaration of Helsinki and was approved by the local ethics committees (1-10-72-558-12). All subjects gave their informed consent to participate prior to the experiments.

### Beverages

During the intervention period, each cyclist received one bottle (750 ml) containing the intervention beverage for every hour of exercise performed. The PRO-CHO group ingested a beverage containing 0.2 g of protein/kg/h (Whey protein hydrolysate with a degree of hydrolysis between 23–29 %, Arla Foods Ingredients Group P/S, Viby, Denmark) and 1 g of CHO/kg/h (Maxim Energy Drink, Maxim International, Ishoej, Denmark). The CHO group consumed an energy-matched carbohydrate beverage containing 1.2 g of CHO/kg/h (Maxim Energy Drink, Maxim International, Ishoej, Denmark). The carbohydrate content in both beverages consisted of maltodextrin and fructose in the ratio 2:1. We chose an isocaloric control beverage rather than a beverage matched for carbohydrate to isolate the effect of the addition of protein rather than an extra amount of energy.

Residual fluid left in used bottles was less than 300 ml in total per training day except for one subject in the PRO-CHO group who was not able to drink the total beverage volume on three of the six days (residual fluid remaining was 400 out of 4500 ml, 600 out of 3750 ml, and 1875 out of 3750 ml, respectively).

Both groups consumed a recovery beverage immediately after exercise each day containing 18 g of protein and 69 g of carbohydrate corresponding to ~0.25 g protein/kg and 1 g carbohydrate/kg (similar sources of protein and carbohydrate as during cycling). Nothing else was ingested, except water, within the two hours before and the one hour after each training session each day. Neither the subjects nor the testing staff was informed about the content of the beverages. Beverages were prepared by staff members not present during the training and performance testing. At D1, the recovery beverage was ingested after the post-exercise testing (D1post).

On Day 6, individual weight changes during the 6 h training session were determined to check hydration status. The athletes were weighed immediately before and after the training session in minimal clothing. The individual bottles were weighed before and after the 6 h training session. On average the athletes were provided with 4.33 L in total during the training session (0.72 L/h). One subject did not consume 0.35 L of the provided beverages during the training session and two subjects consumed additional 0.50 and 0.75 L plain water. Weight data from these three subjects were adjusted in the evaluation of the effect of 0.72 L/h on hydration status (weight change).

### Training schedule and performance testing for the training camp

The training regime was standardized and controlled by the National U23 coach in cycling. The subjects were divided in two groups depending on their training status and history (long: 28.6 h/6 days and short 24.9 h/6 days). The cyclists in the matched pairs were within the same training groups and thereby an equal number of long and short distance subjects were represented in PRO-CHO and CHO respectively. The long distance group biked ~5, 5, 1, 6, 5, and 6 h during the 6 training days (plus standardized warm-up and tests) and the short distance group biked 4, 4, 1, 4, 5, and 6 h (plus standardized warm-up and tests). The training program within both the short and long distance group consisted of a mix of distance training, interval training, mountain climbing, and an individual time trial. The total number of training hours and training distance were identical in the two intervention groups since cyclists in the matched pairs were cycling together.

### Diet control

To avoid any dietary bias, subjects followed a predetermined energy balanced diet plan throughout the week. Prior to the intervention week, each cyclist had an individual meeting with a dietitian where the importance of the diet control was emphasized. Furthermore, food preferences were noted and the subjects were asked if they were suffering from food allergies. Afterwards, an individual diet plan was prepared based on the weight, training load, and preferences using the online software madlogvita (http://www.madlogvita.dk). Total daily energy expenditure (TEE) was estimated for each training day during the intervention and the diet plans varied between days, depending on training schedule. Total daily energy expenditure was estimated based on the following equation: TEE (kJ/day) = RMR x PAL + EX, where RMR is the estimated resting metabolic rate [28], PAL is estimated physical activity level in non-training hours, which was set at 1.5 corresponding to a sedentary lifestyle [28], and EX was the estimated energy used during training sessions (number of training hours × 200 W × 3.6 × (100/25)), assuming an exercise effectiveness of 25 % and an average power output of 200 W. The food was served as a buffet and the participants weighed all the food items in accordance with their individual diet plan. A dietitian was available at the buffet each morning and evening. In addition, participants could always contact and meet with the dietitian if they had a question about their individual diet plans. The subjects were not allowed to consume dietary supplements, sports products, or any food item apart from the items in their individual diet plans.

The average distribution of macronutrients in the diet (excl. intervention beverages) was 8 g of carbohydrate/kg/day (~62 % of energy (E%)) and 1.7 g protein/kg/day. The 18 g protein in the recovery beverage ingested by both groups was included in the 1.7 g protein/kg/day, whereas the protein content in the intervention beverages ingested by PRO-CHO during cycling was in addition to the controlled diet. Fat was supplemented to meet each individual’s energy need (~22–24 E%). The daily intake of protein in CHO corresponded to protein recommendations for elite endurance athletes [29]. The protein intake in PRO-CHO, including the intervention beverages, was 2.6 g/kg/day. Total daily carbohydrate intake including beverages was 14.6 g/kg/day in CHO and 13.6 g/kg/day in PRO-CHO. The energy content of the diet was adjusted (±125 kcal/day) to reestablish energy balance if morning weight had changed more than 1 kg body weight and a bioimpedance measurement did not indicate that the weight change was due to change in hydration. Average energy intake was 24.4 MJ/day in long (29 h training) and 22.0 MJ/day in short (25 h training), including 8.4 MJ/day (long) and 7.2 MJ/day (short) from intervention beverages. The food and nutrient composition of the breakfast before the test protocol at D1 and D6 was the same to ensure standardization of the subjects.

### Determination of VO_2max_

The subjects completed a VO_2max_ test on a stationary bike ergometer (SRM) one or two weeks before the training camp. After a 15-min warm-up, subjects were instructed to work all-out for 5 min during which they were allowed to change load and cadence. VO_2_ was continuously measured through a mask connected to AMIS software (AMIS 2001, Innovision, Odense, Denmark). Every 30 s the average respiratory variables were registered. Heart rate (HR) was continuously recorded using POLAR RS800 or RS800CX. Lactate was measured 1 and 3 min after the test. To ensure that VO_2max_ had been obtained, two of the following four criteria had to be met [30]: 1) VCO_2_/VO_2_ > 1.10, 2) HR was within ±5 beats/min of maximal HR (HR_max_, based on earlier test results), 3) Plateau in oxygen consumption, or 4) Accumulation of lactate (>8 mmol/L).

### Test protocol

The 10-s peak power tests and the 5 min all-out tests were performed on an ergometer bike (Monark Ergomedic model 894E, Monark Exercise AB, Sweden). Within the two weeks before the training camp, the cyclists were familiarized to the test protocol and equipment used during the intervention period. The protocol consisted of a 10-s Peak Power Test, 3 min recovery, followed by a 5-min all-out performance test. The pre-intervention performance test was carried out identically to those during the training camp intervention (same bikes and individual bike settings). The results from the pre-intervention 5-min all-out performance test were used to match the cyclists before block randomization. Blood samples were collected 1 and 3 min after completion of the performance test and analyzed for lactate.

#### 10-s Peak Power Test

Subjects performed a 30-min standardized warm-up program before the pre-intervention, D1pre, and D6pre tests. The warm-up program included 10 min at 75 % HR_max_, 5 min at 80 % HR_max_, 5 min at 75 % HR_max_, 5 min at 90 % HR_max,_ and 5 min at 75 % HR_max._ The D1post test was performed directly after finishing the training session at D1. The 10-s maximal peak power test was conducted at a load corresponding to 10 % of individual body weight. The cyclists were instructed to accelerate to a maximal cadence without any load whereupon they released the load. The subjects were not allowed to rise from the saddle during the test. Peak power and peak power/kg was recorded using the Monark computer software designed for the 894E (Monark anaerobic test software version 3.3.0.0).

#### 5-min all-out performance test

The load during the 5-min all-out performance test was either 3.7 (*n* = 3), 4 (*n* = 7), or 4.3 kg (*n* = 8) depending on the capacity of the cyclist, corresponding to 370, 400, and 430 W if the cadence is 100 rpm. The individual start load was the same in all tests. The subjects accelerated to 100 rpm without load, after which, the load was added and the test was started. The cyclists were informed to achieve as high an average watt as possible during the 5-min all-out test. The load was adjusted by the testing staff if the cyclist gave sign to increase or reduce the load. The total work performed and the average power was calculated using the same software program as above.

The cyclists in the matched pairs performed the tests simultaneously at D1pre, D1post, and D6pre.

### Biochemical analysis

Venous blood samples were immediately centrifuged and frozen. Samples were analyzed for markers of muscle damage (CK, LDH, and myoglobin) and for cortisol at the Department of Bioscience, Aarhus University Hospital. Coefficient of variation (CV) for CK, LDH, myoglobin, and cortisol determination was 2.8–3.9 %, 3.0–4.0 %, 6.8–9.0 %, and 5.9–6.0 %, respectively, depending on the concentration level.

Capillary blood was obtained by finger sticks and immediately analyzed for lactate by an enzymatic lactate analyzer (Yellow Springs Instruments lactate analyzer model 23 L).

Saliva samples were analyzed for IgA with a Salivary Secretory IgA Enzyme Immunoassay kit (Salimetrics Europe, Ltd., Item No. 1–1602, Suffolk, UK) using a BioRad Model 550 Microplate Reader and Microplate Manager version 5.2.1. Morning saliva samples were collected during a 3-min period and post-exercise samples during 2 min. The analytic results were corrected for differences in saliva flow rate by multiplying the results by the saliva produced during the collection period (flow rate: ml min^−1^).

### Statistical analysis

Data were log-transformed when appropriate based on an inspection of the standardized residuals (CK, LDH, cortisol). Overall, performance data, CK, LDH, myoglobin, cortisol, IgA, and weight were analyzed using multivariate repeated measurements ANOVA with treatment (PRO-CHO and CHO) and time interactions, followed by Student-Newman-Keuls Post-hoc test if significance was observed. The unequal standard deviations and correlations in the two groups were taken into account in all the analyses by letting the standard deviations and correlations vary between groups. In more detail, the performance data was tested separately for an acute effect (D1pre vs D1post) and for a long-term effect (D1pre vs D6pre). Data for body weight and IgA was analyzed for an acute effect on D6 (before and after training the training session). The CK, LDH, myoglobin, cortisol, IgA, and body weight data were tested for a long-term effect (D1 to D7), both for interaction and time effect as well as separated one-way ANOVA analysis for the PRO-CHO and the CHO groups if interaction effect was significant.

The data was analyzed using Stata version 12.1. P values set at <0.05 were taken to indicate statistical significance. Data is reported as means ± standard error of the mean (SE) if nothing else is stated.

## Results

### Performance

**Fig. 2:**
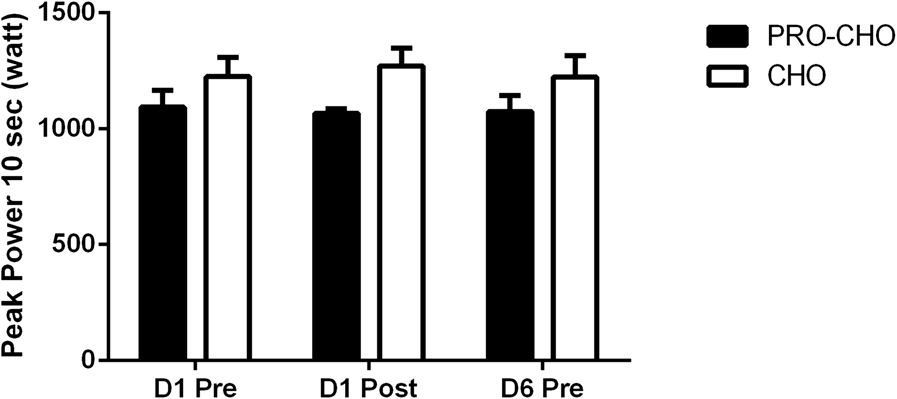
Peak power output during a 10-s sprint test completed on the first day of the training camp before (D1pre), after the first training session (D1post), and before the training session in the morning of Day 6 (D6pre). Data are shown as means ± SE

Peak power output for the 10-s test at D1-pre was 1224 ± 72 W and 1094 ± 84 W in CHO and PRO-CHO, respectively (*p* = 0.25), and did not change significantly in D1-post and D6-pre in either group (Fig. 2). Similarly, there was no significant difference in peak power expressed per kg of body weight between groups at baseline (17 ± 1 and 15 ± 1 W/kg in CHO and PRO-CHO, respectively, *p* = 0.16), and no significant change was observed over time or between groups.

**Fig. 3:**
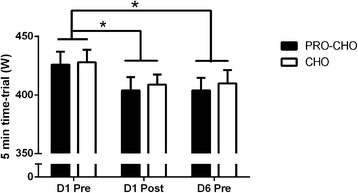
Five-minute all-out performance test completed on the first day before (D1pre) and after (D1post) the training session as well as on Day 6 before the training session (D6pre). Data are shown as means ± SE. * *p* < 0.01: Significant time effect between D1pre and D1post and between D1pre and D6pre

The average power during the 5-min all-out performance test at D1-pre was 426 ± 11 W and 428 ± 11 W in CHO and PRO-CHO, respectively (*p* = 0.88) (Fig. 3). The average power output was lower (*p* < 0.01) after training at D1 (D1-post) in both CHO and PRO-CHO (409 ± 8 W and 404 ± 11 W) as well as in the morning of D6 (410 ± 11 W and 404 ± 11 W) (Fig. 3).

### Markers of muscle damage

**Fig. 4:**
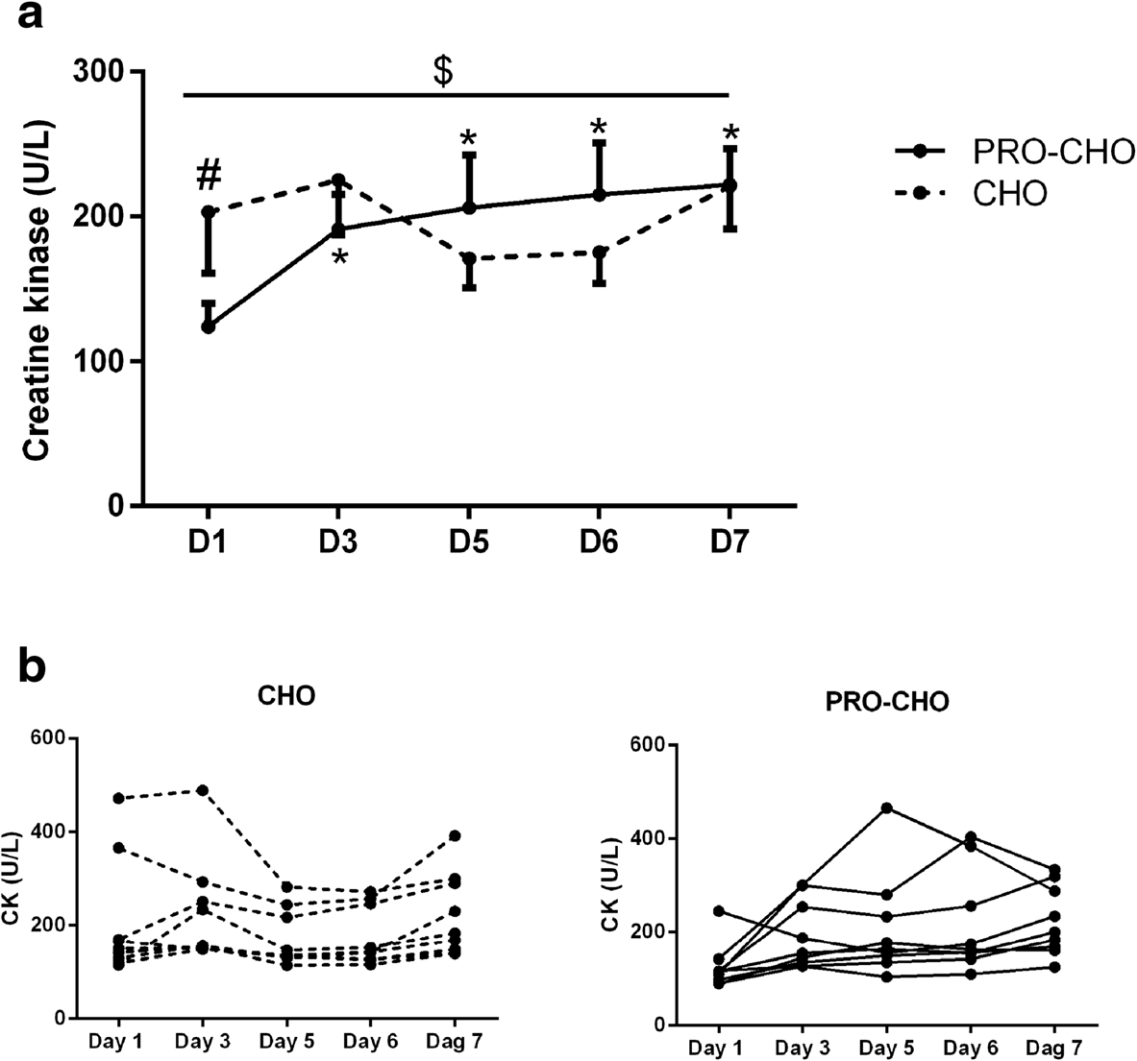
Plasma creatine kinase (CK) measured in the morning at Day 1, 3, 5, 6, and 7. **a** Mean values of CK in the two groups (means ± SE); **b** Individual changes in CK in each group. # *p* < 0.05: Significant difference between groups (PRO-CHO vs. CHO) at baseline at D1. * *p* < 0.05: Significant difference from D1 in the PRO-CHO group. $ *p* < 0.01: Overall significant interaction between time and treatment effect

At D1 before the intervention, CK was higher (~63 %, p < 0.05) in CHO (203 ± 42.2) than in PRO-CHO (124 ± 16.0, *p* < 0.05). An overall change in CK during the six training days was observed (*p* < 0.01), which was influenced by treatment (interaction (*p* < 0.01; Fig. 4). In PRO-CHO, CK was higher in the morning of D3, D5, D6, and D7 compared to D1 (*p* < 0.05). In contrast, in CHO no significant change was observed compared to baseline (Fig. 4).

**Fig. 5:**
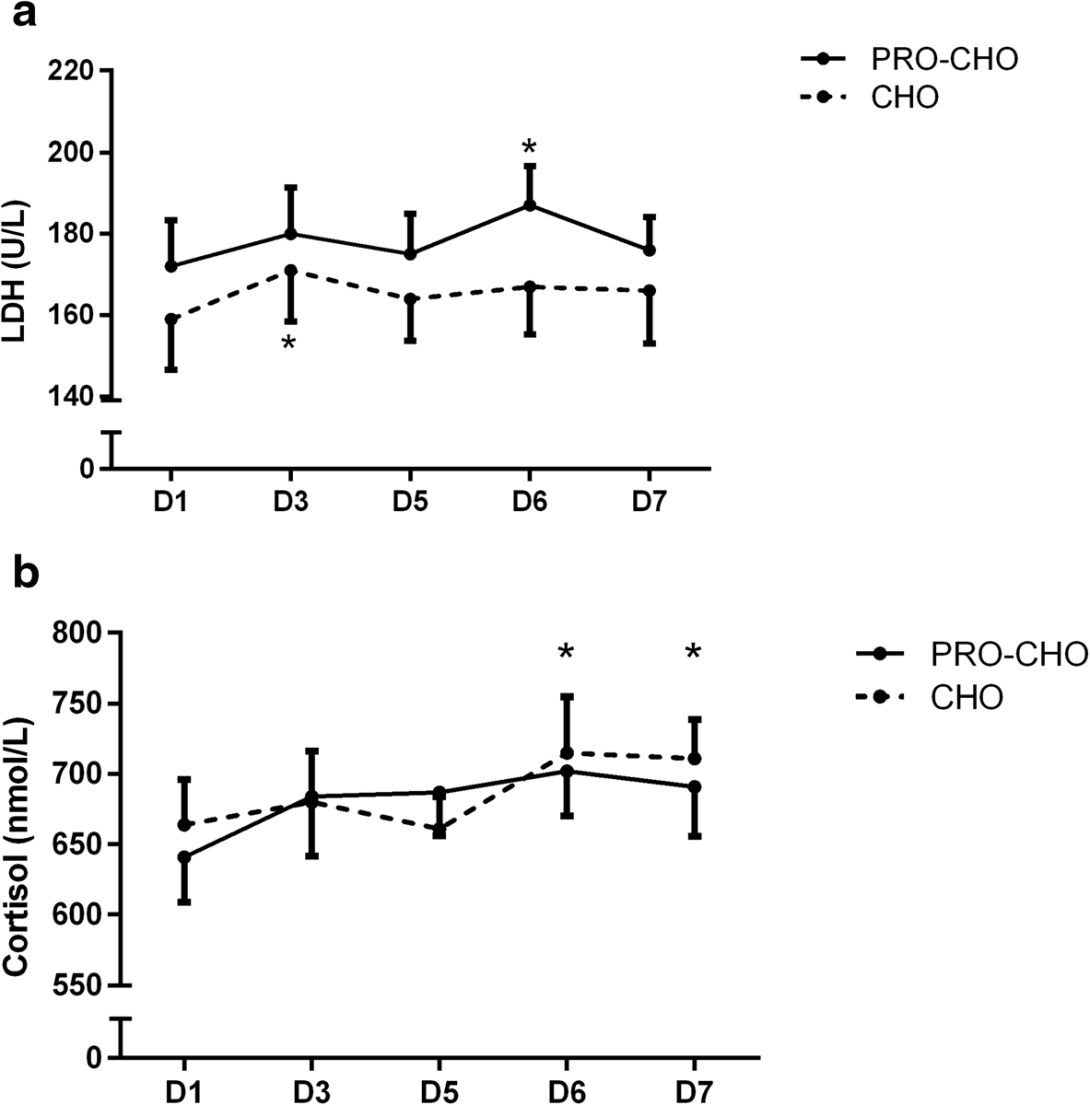
Plasma lactate dehydrogenase (LDH)(**a**) and cortisol (**b**) measured in the morning at day 1, 3, 5, 6, and 7. Data are shown as means ± SE. * *p* < 0.05: Significant difference from baseline within groups

LDH was not significantly different between groups at baseline (159 ± 12 U/L and 172 ± 11 U/L in CHO and PRO-CHO, respectively; *p* = 0.44) and there was no interaction between treatment (PRO-CHO vs. CHO) and development of LDH over time (*p* = 0.92). Nevertheless, LDH increased over time (*p* < 0.01). The level of LDH was higher at D3 compared to D1 within CHO (*p* < 0.01) and at D6 compared to D1 in PRO-CHO (*p* < 0.05; Fig. 5a).

Plasma myoglobin was below detection level (<53 μg L^−1^) in all morning samples except for one subject in PRO-CHO, where myoglobin level reached 73 μg L^−1^ at D3 (data not shown).

### Markers of stress and immune function

In the morning samples, cortisol increased over time (*p* < 0.001) and was significantly higher at D6 and D7 compared to Day 1, but no significant interaction effect was observed (*p* = 0.50; Fig. 5b).

**Fig. 6:**
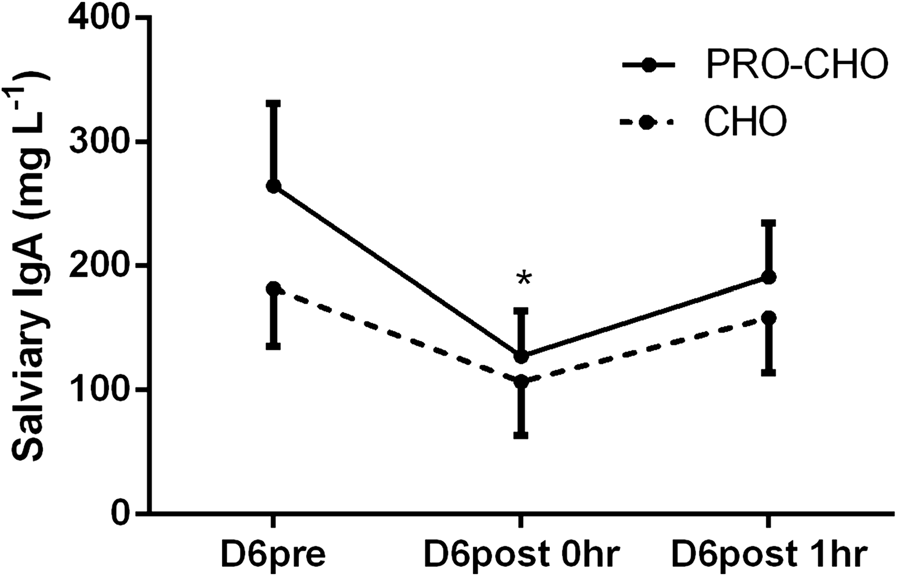
Salivary IgA before (D6pre), immediately after (D6post 0 h), and one hour (D6post 1 h) after the six-hour training session at Day 6. Data are shown as means ± SE. * *p* < 0.05: Salivary IgA higher D6pre compared to D6post 0 h

Salivary IgA was significantly lower immediately post-exercise at D6 (*p* < 0.05) compared to the morning sample at D6, but there was no significant difference between groups in the development of salivary IgA (interaction, *p* = 0.64; Fig. 6). In the morning samples, salivary IgA was not significantly changed during the training camp (*p* = 0.36) and no interaction was observed (*p* = 0.26; data not shown).

### Body weight

Body weight measured after overnight fasting did not change significantly from the first to the last day of the training camp in PRO-CHO (−0.3 ± 0.8 kg) or CHO (−0.6 ± 1.0 kg). During the six-hour training session at D6 the cyclists consumed 0.72 L/h (in total ~4.3 L). Nevertheless, the body weight in PRO-CHO was significantly lower after the training session at D6 (71.5 ± 2.3 kg) compared with immediately before the training session (72.1 ± 1.4 kg, *p* < 0.05), whereas no significant difference was observed in CHO (71.6 ± 1.3, vs. 71.9 ± 2.5 kg, *p* = 0.17). However, the change in body weight was not significantly different between groups (−0.8 ± 0.3 % vs. -0.4 ± 0.3 % in PRO-CHO and CHO, respectively, *p* = 0.41).

## Discussion

The novelty of the present study is that the effect of protein ingestion during exercise was evaluated in young (U23) top-class cyclists under real life training conditions with a strict diet control. Subjects ingesting CHO during the training sessions followed recommended protein intake (1.7 g protein/kg/day), whereas the PRO-CHO ingested an additional 0.9 g protein/kg/day. Not only was the acute effect of protein ingestion examined, but also the accumulated effects during a strenuous training camp (25–29 cycling hours within 6 days). The main findings of the present study were that compared to isocaloric ingestion of carbohydrate, partial substitution of carbohydrates with whey protein hydrolysate during exercise did not significantly reduce the decline in performance after 4–5 h cycling or after five days of strenuous training. Furthermore, the intake of protein during training did not have a marked influence on the changes in markers of muscle damage (CK and LDH), cortisol, or immune function (salivary IgA) during the training camp.

### Acute effects of ingestion of protein on performance

Potential positive effects of protein ingestion, such as glycogen sparing, maintaining Krebs cycle intermediates, or reducing central fatigue [31], have led to the hypothesis that protein ingestion during exercise would improve endurance performance. We observed no acute effect of performance on a subsequent 10-s sprint test and the 5-min performance test after consumption of 14 g protein/h during 4 or 5 h cycling compared to consumption of isocaloric carbohydrate beverages. This is in line with several other studies showing no advantage of adding protein to carbohydrate beverages during exercise [3, 5, 6, 32, 33]. Our trial adds support to this general observation by providing new data based on elite endurance cyclists. Studies that have found an acute performance effect of protein supplementation have either been non-isocaloric [9, 7, 10] or have examined performance effects during heat stress [8]. The importance of comparing isocaloric beverages rather than non-isocaloric beverages (matched for carbohydrate content) ingested during exercise on endurance capacity is underlined by results from two studies from the same research group using almost identical protocols [9, 15]. When using an isocaloric control beverage no difference in TTE was observed when partly substituting carbohydrate with protein [15]. In contrast, when effects of beverages matched for carbohydrate content was studied, TTE was improved (29 %, 106 vs 82 min) during cycling when protein was added to the beverage [9]. The latter finding may be a consequence of either a 20 % higher energy intake when protein was added or a suboptimal carbohydrate intake in both groups (~38 g/h). When the carbohydrate intake during endurance exercise is in accordance with the recommendations [1, 2, 29], as in our present trial, no acute beneficial effect on TT [3, 34] or TTE [6, 15, 35–37] of either partial substitution of carbohydrate with protein or adding protein to the carbohydrate supplement is reported.

### Long-term effects of ingestion of protein during exercise on performance

Even if protein ingestion during exercise does not have an acute beneficial effect on performance when the recommended amount of carbohydrate is ingested, it could have a positive effect on subsequent recovery and performance. Optimizing recovery is especially important for elite cyclists who often have to compete several days in a row. We observed a decline in performance at Day 6 compared to before initiating the intervention, but the decrease in performance was not significantly different between groups as hypothesized. This finding is in line with studies of moderately or well-trained subjects where protein along with carbohydrate ingested during and immediately after exercise compared to carbohydrate beverages show no effect on TTE [15] or TT [38] performed less than 24 h after exercise completion. Nevertheless, in a randomized cross-over trial, 10 well-trained cyclists (66.2 ± 6 mL O_2_/kg/min) experienced improved performance (1.8 %) in a TT (~20 k) after 4 h of recovery when the athletes had ingested a PRO-CHO beverage (0.33 g/kg/h + 0.87 g/kg/h) vs isocaloric CHO beverage (1.2 g/kg/h) during a 2.5 h cycling bout followed by a PRO-CHO recovery beverage immediately after the exercise session [11]. The content of the beverages ingested during the initial exercise bout was in accordance with the beverages used in the present study, but the discrepancy in theirs [11] our findings is likely related to either the duration of the recovery period between exercise sessions or the type of test. In the majority of road bike races, an overnight recovery is possible. The prolonged overnight recovery may render any benefits of protein ingestion during exercise inconsequential compared to shorter recovery duration.

The athletes in the present study were supplied 18 g protein/CHO immediately after the training sessions as part of their normal routine, and we aimed to investigate whether additional protein during the training session could prevent the gradual decrease in performance during repeated days of strenuous cycling during a training camp. In contrast to our hypothesis, performance in the 5-min “all-out” test declined similarly in CHO and PRO-CHO during the 6 days of training camp, whereas 10-s sprint performance did not decline. The effect of combined CHO-PRO vs CHO supplementation on cycling performance during eight days of a strenuous mountain bike race in a hot environment has been studied in a previous study [20]. In this study, the PRO-CHO supplemented group completed the race faster than the CHO supplemented group, but in contrast to our study, ingestion of the beverages was not controlled but ingested *ad libitum*. Furthermore, the daily total energy content was not controlled, and in the CHO group, the body weight was reduced after the intervention period. Therefore, the performance-enhancing effect of PRO-CHO compared to CHO supplementation during repeated days of racing in the previous trial [20] may be related to negative energy balance in the CHO group rather than the addition of protein to the carbohydrate beverage.

In the present trial the diet was energy-balanced, standardized, and controlled. The daily protein intake in the control group (CHO) was 1.7 g/kg, corresponding to recommendations for elite endurance athletes [39–40]. A carbohydrate-rich diet was served to both groups to negate any effect of suboptimal carbohydrate intake on recovery of glycogen stores between training sessions and a recovery-beverage containing both carbohydrate and protein was served immediately after each training session in line with the recommendation and normal practice within this group of elite cyclists. Therefore, we cannot rule out that protein ingestion during exercise would be beneficial on performance under circumstances where the nutrient guidelines are not followed and/or the energy intake is insufficient.

### Test protocol

The acute and accumulated reduction in 5-min all-out performance during the training camp reflected the high physical demand of the camp. A time trial test was chosen rather than a time to exhaustion test because time trial tests more closely simulate an actual performance situation and hence are regarded a more valid measure of performance [41]. Nevertheless, the performance in the 10-s sprint test was not significantly affected after either 4–5 h cycling or the accumulated hours of cycling during the training camp. The latter indicates that this test was not sensitive to catch development of fatigue and impairment in recovery. The 10-s peak power test was chosen as peak power is typically obtained after 3–5 s in a 30-s Wingate test [42], and this test allows quick recovery with the impending 5-min performance test in mind.

### Markers for muscle damage

LDH and myoglobin as markers for muscle damage were not significantly different between the groups. Surprisingly, the results suggest that carbohydrate supplementation rather than protein plus carbohydrate attenuated the increase in plasma concentration of CK as a marker for muscle damage during the strenuous training camp. However, there was a large variation in CK between the subjects and the overall CK values were at the low end. Additionally, it must be emphasized that CK was higher at baseline in the CHO group due to high values in two subjects. Therefore, one should be cautious when drawing conclusions. Overall, the changes in markers for muscle damage were small, which probably is explained by the fact that cycling is primarily based on concentric muscle contractions. In agreement with the latter suggestion, orienteering has a greater proportion of eccentric muscle actions, and in a group of elite orienteers we in an earlier study observed an increase in markers for muscle damage in the group ingesting carbohydrates before and after each of 13 training sessions during a one-week training camp [21]. This increase was attenuated in the group ingesting protein before and a protein-carbohydrate beverage after each training session. Although not all trials are in agreement [20, 40, 43], many previous trials [15, 17, 19, 21, 23, 44] show that protein ingestion immediately *after* exercise attenuates markers of muscle damage. Therefore, the addition of a protein supplement *during* exercise may further attenuate the increased rate of muscle damage that is associated with exercise that includes an eccentric component. Future trials should clarify if protein supplementation *during* exercise has an additive effect for decreasing muscle damage beyond what has been observed when provided immediately *after* exercise.

### Immune function

Studies have shown that high availability of carbohydrate reduces the immune depression associated with extreme endurance training in athletes [45]. However, little is known about the effect of protein supplementation on immune function in relation to exercise [45]. In the present study, changes in the immune marker salivary IgA did not differ between groups, which may be related to the fact that subjects were in energy balance and the carbohydrate intake was high in both groups.

### Limitations

A drawback of the present study was that we were not allow to obtain muscle biopsies from the subjects since they were all part of or candidates for the Danish National Team in cycling (U23). Analysis of muscle biopsies could have given information about if the adaptation to training stimuli differed between the intervention groups.

This field study was performed at a training camp with a temporarily established laboratory. Since the time frame for testing all athletes in the beginning and end of training camp was very short, it was only possible to perform a short-term performance test. We assumed that extra protein ingested during training could reduce muscle damage and this would be reflected in a short-term test. Nevertheless, we cannot rule out that ingestion of extra carbohydrate during cycling in CHO compared to PRO-CHO actually would be beneficial for performance in a more long-term endurance test due to greater glucose availability.

It is debatable whether our results are transferable to other groups of elite endurance athletes than cyclists. Cycling is dominated by concentric muscle contraction and probably only a minimum of muscle damage is induced, even though the cyclists increased their training load during the training camp. We cannot exclude that other types of athletes (runners and triathletes) may benefit from ingestion of protein during exercise when following a strenuous training regime, particularly those that use predominantly eccentric muscle contractions (such as downhill running and resistance training).

A recovery beverage was provided after each training session with a dose of protein corresponding approximately to the amount of high-quality protein that appears to maximize the acute anabolic response in skeletal muscle [46]. We hypothesize that the recovery beverage may have diminished the potential positive effect of ingestion of protein during exercise. Therefore, it is possible that we would have observed a positive effect in PRO-CHO if the habitual recovery beverage had been excluded during the training camp. In regards to mitigating markers of muscle damage, a potential positive effect of co-ingestion of protein during exercise may have been visible if we had included elderly subjects who require a greater amount of protein (35–40 g protein) to maximize skeletal muscle protein synthesis rates [47, 48].

## Conclusion

Partial substitution of carbohydrate with whey protein hydrolysate during exercise has no significant acute ergogenic effect on performance in elite cyclists compared to ingestion of isocaloric recommended amounts of carbohydrates. Similarly, when a recovery drink containing PRO + CHO was ingested immediately post-exercise, the decline in performance after a strenuous training camp was not significantly different between the groups, indicating no beneficial effect on recovery from ingesting protein plus carbohydrate during exercise compared to ingestion of carbohydrate alone.

## Abbreviations

CHO: Carbohydrate
CK: Creatine kinase
E%: Energy %
HR_max_: Maximal heart rate
IgA: Immunoglobulin
LDH: Lactate dehydrogenase
OAL: Physical activity level
PRO-CHO: Protein and carbohydrate
TT: Time trial
TTE: Time to exhaustion
TEE: Total energy expenditure

## References

[1] Burke LM, Hawley JA, Wong SH (2011). Carbohydrates for training and competition. J Sports Sci.

[2] Cermak NM, van Loon LJ (2013). The use of carbohydrates during exercise as an ergogenic aid. Sports Med.

[3] Breen L, Tipton KD, Jeukendrup AE (2010). No effect of carbohydrate-protein on cycling performance and indices of recovery. Med Sci Sports Exerc.

[4] Cheuvront SN, Carter R, Kolka MA (2004). Branched-chain amino acid supplementation and human performance when hypohydrated in the heat. J Appl Physiol.

[5] Madsen K, MacLean DA, Kiens B (1996). Effects of glucose, glucose plus branched-chain amino acids, or placebo on bike performance over 100 km. J Appl Physiol (1985).

[6] Osterberg KL, Zachwieja JJ, Smith JW (2008). Carbohydrate and carbohydrate + protein for cycling time-trial performance. J Sports Sci.

[7] Ivy JL, Res PT, Sprague RC (2003). Effect of a carbohydrate-protein supplement on endurance performance during exercise of varying intensity. Int J Sport Nutr Exerc Metab.

[8] Mittleman KD, Ricci MR, Bailey SP (1998). Branched-chain amino acids prolong exercise during heat stress in men and women. Med Sci Sports Exerc.

[9] Saunders MJ, Kane MD, Todd MK (2004). Effects of a carbohydrate-protein beverage on cycling endurance and muscle damage. Med Sci Sports Exerc.

[10] Saunders MJ, Luden ND, Herrick JE (2007). Consumption of an oral carbohydrate-protein gel improves cycling endurance and prevents postexercise muscle damage. J Strength Cond Res.

[11] Hall AH, Leveritt MD, Ahuja KD (2013). Coingestion of carbohydrate and protein during training reduces training stress and enhances subsequent exercise performance. Appl Physiol Nutr Metab.

[12] Berardi JM, Noreen EE, Lemon PW (2008). Recovery from a cycling time trial is enhanced with carbohydrate-protein supplementation vs Isoenergetic carbohydrate supplementation. Int Soc Sports Nutr.

[13] Betts J, Williams C, Duffy K (2007). The influence of carbohydrate and protein ingestion during recovery from prolonged exercise on subsequent endurance performance. J Sports Sci.

[14] Goh Q, Boop CA, Luden ND (2012). Recovery from cycling exercise: Effects of carbohydrate and protein beverages. Nutrients.

[15] Romano-Ely BC, Todd MK, Saunders MJ (2006). Effect of an isocaloric carbohydrate-protein-antioxidant drink on cycling performance. Med Sci Sports Exerc.

[16] Hill KM, Stathis CG, Grinfeld E (2013). Co-ingestion of carbohydrate and whey protein isolates enhance pgc-1alpha mrna expression: A randomised, single blind, cross over study. Int Soc Sports Nutr.

[17] Nelson AR, Phillips SM, Stellingwerff T (2012). A protein-leucine supplement increases branched-chain amino acid and nitrogen turnover but not performance. Med Sci Sports Exerc.

[18] Luden ND, Saunders MJ, Pratt CA (2006). Effects of a six-day carbohyydrate/protein intervention on muscle damage, soreness, and performance in runners. Med Sci Sports Exerc.

[19] Skillen RA, Testa M, Applegate EA (2008). Effects of an amino acid carbohydrate drink on exercise performance after consecutive-day exercise bouts. Int J Sport Nutr Exerc Metab.

[20] Cathcart AJ, Murgatroyd SR, McNab A (2011). Combined carbohydrate-protein supplementation improves competitive endurance exercise performance in the heat. Eur J Appl Physiol.

[21] Hansen M, Bangsbo J, Jensen J, et al. Effect of whey protein hydrolysate on performance and recovery of top-class orienteering runners. Int J Sport Nutr Exerc Metab. 2014. 10.1123/ijsnem.2014-0083 25029703

[22] Rowlands DS, Rossler K, Thorp RM (2008). Effect of dietary protein content during recovery from high-intensity cycling on subsequent performance and markers of stress, inflammation, and muscle damage in well-trained men. Appl Physiol Nutr Metab.

[23] Thomson JS, Ali A, Rowlands DS (2011). Leucine-protein supplemented recovery feeding enhances subsequent cycling performance in well-trained men. Appl Physiol Nutr Metab.

[24] Lollo PCB, Amaya-Farfan J, Faria IC (2014). Hydrolysed whey protein reduces muscle damage markers in brazilian elite soccer players compared with whey protein and maltodextrin. A twelve-week in-championship intervention. Int Dairy J.

[25] Ferguson-Stegall L, McCleave EL, Ding Z (2011). Postexercise carbohydrate-protein supplementation improves subsequent exercise performance and intracellular signaling for protein synthesis. J Strength Cond Res.

[26] Meeusen R, Duclos M, Foster C (2013). Prevention, diagnosis, and treatment of the overtraining syndrome: Joint consensus statement of the european college of sport science and the american college of sports medicine. Med Sci Sports Exerc.

[27] Durnin JV, Womersley J (1974). Body fat assessed from total body density and its estimation from skinfold thickness: Measurements on 481 men and women aged from 16 to 72 years. Br J Nutr.

[28] MINISTERS NCO, Nordic nutrition recommendations nnr 2004, integrating nutrition and physical activity., Ministers. NCo, Editor. 2005: Copenhagen:.

[29] American Dietetic A, Dietitians of C, American College of Sports M (2009). American college of sports medicine position stand. Nutrition and athletic performance. Med Sci Sports Exerc.

[30] Stachenfeld NS, Eskenazi M, Gleim GW (1992). Predictive accuracy of criteria used to assess maximal oxygen consumption. Am Heart J.

[31] Newsholme EA, Blomstrand E (2006). Branched-chain amino acids and central fatigue. J Nutr.

[32] Cheuvront SN, Carter R, Kolka MA (2004). Branched-chain amino acid supplementation and human performance when hypohydrated in the heat. J Appl Physiol.

[33] Greer BK, Woodard JL, White JP (2007). Branched-chain amino acid supplementation and indicators of muscle damage after endurance exercise. Int J Sport Nutr Exerc Metab.

[34] van Essen M, Gibala MJ (2006). Failure of protein to improve time trial performance when added to a sports drink. Med Sci Sports Exerc.

[35] Martinez-Lagunas V, Ding Z, Bernard JR (2010). Added protein maintains efficacy of a low-carbohydrate sports drink. J Strength Cond Res.

[36] Valentine RJ, Saunders MJ, Todd MK (2008). Influence of carbohydrate-protein beverage on cycling endurance and indices of muscle disruption. Int J Sport Nutr Exerc Metab.

[37] van Hall G, Raaymakers JS, Saris WH (1995). Ingestion of branched-chain amino acids and tryptophan during sustained exercise in man: Failure to affect performance. J Physiol.

[38] Cermak NM, Solheim AS, Gardner MS (2009). Muscle metabolism during exercise with carbohydrate or protein-carbohydrate ingestion. Med Sci Sports Exerc.

[39] Phillips SM, Van Loon LJ (2011). Dietary protein for athletes: From requirements to optimum adaptation. J Sports Sci.

[40] Burke L, et al. Clinical sports nutrition: McGraw-Hill Companies; 2006 [Burke LM and Deakin V (Series Editor), vol Third edition.]

[41] Currell K, Jeukendrup AE (2008). Validity, reliability and sensitivity of measures of sporting performance. Sports Med.

[42] Driss T, Vandewalle H (2013). The measurement of maximal (anaerobic) power output on a cycle ergometer: A critical review. Biomed Res Int.

[43] Rowlands DS, Wadsworth DP (2011). Effect of high-protein feeding on performance and nitrogen balance in female cyclists. Med Sci Sports Exerc.

[44] Luden ND, Saunders MJ, Todd MK (2007). Postexercise carbohydrate-protein- antioxidant ingestion decreases plasma creatine kinase and muscle soreness. Int J Sport Nutr Exerc Metab.

[45] Gunzer W, Konrad M, Pail E (2012). Exercise-induced immunodepression in endurance athletes and nutritional intervention with carbohydrate, protein and fat-what is possible, what is not?. Nutrients.

[46] Moore DR, Robinson MJ, Fry JL (2009). Ingested protein dose response of muscle and albumin protein synthesis after resistance exercise in young men. Am J Clin Nutr.

[47] Yang Y, Breen L, Burd NA (2012). Resistance exercise enhances myofibrillar protein synthesis with graded intakes of whey protein in older men. Br J Nutr.

[48] Pennings B, Groen B, de Lange A (2012). Amino acid absorption and subsequent muscle protein accretion following graded intakes of whey protein in elderly men. Am J Physiol Endocrinol Metab.

